# Evaluation of pediatric patients presenting with acute-onset unilateral transient acquired blepharoptosis

**DOI:** 10.5935/0004-2749.20220082

**Published:** 2025-02-11

**Authors:** Serkan Kirik, Furkan Kirik, Nursel Yurttutan, Olcay Gungor, Can Acipayam

**Affiliations:** 1 Department of Pediatric Neurology, Faculty of Medicine, Kahramanmaras Sutcu Imam University, Kahramanmaras, Turkey; 2 Department of Ophthalmology, Faculty of Medicine, Bezmialem Vakif University, Istanbul, Turkey; 3 Department of Radiology, Faculty of Medicine, Kahramanmaras Sutcu Imam University, Kahramanmaras, Turkey; 4 Department of Pediatric Hematology and Oncology, Faculty of Medicine, Kahramanmaras Sutcu Imam University Kahramanmaras, Turkey

**Keywords:** Blepharoptosis, Craniocerebral trauma, Infectious disease, Miller Fisher syndrome, Horner syndrome, Child, Blefaroptose, Trauma craniocerebral, Síndrome de Miller Fisher, Síndrome de Horner, Criança

## Abstract

**Purpose:**

To evaluate the clinical features of pe diatric patients with acute-onset, unilateral transient acquired blepharoptosis.

**Methods:**

In this retrospective study, the clinical records of patients between April 2015 and June 2020 were reviewed for evaluation of demographic features, accompanying neurological and ophthalmologic manifestations, symptom duration, etiological cause, and imaging findings. Patients with congenital and acquired blepharoptosis with chronic etiologies were excluded.

**Results:**

Sixteen pediatric patients (10 boys and 6 girls) with acquired acute-onset unilateral transient blepharoptosis were included in this study. The patients’ mean age was 6.93 ± 3.16 years. The most commonly identified etiological cause was trauma in 7 patients (43.75%) and infection (para-infection) in 5 patients (31.25%). In addition, Miller Fisher syndrome, Horner syndrome secondary to neuroblastoma, acquired Brown’s syndrome, and pseudotumor cerebri were identified as etiological causes in one patient each. Additional ocular findings accompanied blepharoptosis in 7 patients (58.33%). Blepharoptosis spontaneously resolved, without treatment, in all the patients, except those with Miller Fisher syndrome, neuroblastoma, and pseudotumor cerebri. None of the patients required surgical treatment and had ocular morbidities such as amblyopia.

**Conclusion:**

This study demonstrated that acute-onset unilateral transient blepharoptosis, which is rare in childhood, may regress without the need for surgical treatment in the pediatric population. However, serious pathologies that require treatment may present with blepharoptosis.

## Abstract (pt)

**Objetivo:**

Avaliar as características clínicas de pacientes pediátricos com blefaroptose adquirida unilateral, transitória e de início agudo.

**Métodos:**

Neste estudo retrospectivo, foram revisados prontuários clínicos entre abril de 2015 e junho de 2020. Os pacientes foram avaliados em termos de características demográficas, manifestações neurológicas e oftalmológicas associadas, duração dos sintomas, etiologia e achados de imagem. Foram excluídos pacientes com blefaroptose congênita e com blefaroptose adquirida de etiologia crônica.

**Resultados:**

Foram incluídos neste estudo 16 pacientes pediátricos (10 masculinos e 6 femininos) com blefaroptose adquirida transitória unilateral de início agudo. A média de idade dos pacientes foi de 6,93 ± 3,16 anos. As causas etiológicas mais comumente identificadas foram trauma em 7 pacientes (43,75%) e infecção (casos parainfecciosos) em 5 pacientes (31,25%). Além disso, a síndrome de Miller-Fisher, a síndrome de Horner secundária a neuroblastoma, a síndrome de Brown adquirida e pseudotumor cerebral foram determinados como causas etiológicas em um paciente cada uma. Achados oculares adicionais estavam associados à blefaroptose em 7 pacientes (58,33%). Foi observada a resolução espontânea da blefaroptose, sem tratamento, em todos os pacientes, exceto nos pacientes com síndrome de Miller-Fisher, neuroblastoma e pseudotumor cerebral. Nenhum paciente precisou de tratamento cirúrgico. Morbidades oculares, como ambliopia, não foram encontradas em nenhum paciente.

**Conclusão:**

Este estudo demonstrou que a blefaroptose transitória unilateral de início agudo, rara na infância, pode regredir sem a necessidade de tratamento cirúrgico na população pediátrica. No entanto, também não deve ser esquecido que patologias graves que requerem tratamento podem se apresentar com blefaroptose.

## INTRODUCTION

Blepharoptosis (or ptosis) is the drooping of the upper eyelid caused by functional loss of the muscles and/or nerves that regulate the elevation of the upper eyelid^(1)^. Etiological causes vary according to age groups. Unlike in the adult population, unless treated, blepharoptosis can cause permanent visual complications such as amblyopia in the early pediatric age. If the visual axis is permanently obstructed, early surgery is recommended^(2,3)^. Congenital blepharoptosis is the most common type in pediatric patients. Acquired blepharoptosis is less common in the pediatric population and has been rarely evaluated in studies^(1,4-10)^. Only few studies have identified various etiological causes underlying acquired pediatric ptosis. Early management of the causes of stable or progressive blepharoptosis is important to prevent possible visual complications. However, some causes of acquired blepharoptosis have a good prognosis with spontaneous resolution^(11)^. Therefore, recognition of the clinical features of pediatric patients with transient blepharoptosis is important for preventing unnecessary surgeries or complications such as amblyopia, which occurs due to delayed treatment.

The aim of this study was to evaluate the clinical features of pediatric patients with acute-onset, unilateral transient acquired blepharoptosis.

## METHODS

We retrospectively reviewed the medical records of pediatric patients with acquired ptosis who were admitted to the pediatric neurology department of a tertiary hospital between April 1, 2016, and May 30, 2020. Patients with newly diagnosed acute-onset transient blepharoptosis were included in the study. The patients were evaluated in terms of age and sex, accompanying neurological and ophthalmologic findings, symptom duration, history of infectious disease, and imaging findings. The patient records were evaluated in terms of age and sex, clinical history, duration of ptosis, abnormal findings detected using diagnostic tools such as laboratory and imaging methods, neuro-ophthalmologic findings, other system findings, and presence of chronic disease. Patients with congenital ptosis, acquired blepharoptosis with chronic underlying diseases (e.g., myasthenia gravis and chronic progressive external ophthalmoplegia), or previously known systemic disease were excluded from the study.

Statistical analysis was performed using the SPSS software (Chicago, IL) for Windows version 22. All quantitative data were expressed as mean ± standard deviation. All categorical variables are expressed as number and percentage (n, %). The study approval was obtained from the local institutional ethics committee. Informed consent was obtained from the parents of all patients. The study was performed in accordance with the Declaration of Helsinki.

## RESULTS

Sixteen pediatric patients who met the identified criteria were included in the study. Ten patients (62.25%) were male, and 6 (37.5%) were female. The patients’ mean age was 6.59 ± 2.97 years (minimum: 28 months, maximum: 13 years). Eight patients had right-sided blepharoptosis, and 8 had left-sided blepharoptosis. No bilateral involvement was encountered. The demographic and clinical characteristics of the patients are summarized in table 1.

**Table 1:** Demographic, clinical, and etiological characteristics of the study population

Case no:	Age	Sex	Ocular findings	Etiology	Time to recovery (days)
1	6 years	M	Ptosis	Trauma	3
2	4 years	M	Inferior rectus palsy + ptosis	Trauma	7
3	7 years	F	Ptosis	Trauma	4
4	13 years	M	Superior rectus palsy + ptosis	Trauma	6
5	9 years	F	Ptosis	Trauma	5
6	7 years	M	Ptosis	Trauma	5
7	28 months	M	Ptosis	Trauma	6
8	5 years	M	Ptosis	Infection	4
9	8 years	M	Ptosis	Infection	2
10	5 years	F	Ptosis	Infection	5
11	7 years	M	Ptosis	Infection	4
12	10 years	F	Medial rectus palsy + ptosis	Infection	6
13	30 months	F	Superior rectus palsy + ptosis	MFS	9
14	12 years	M	Ptosis + myosis + papilledema	Horner Syndrome secondary to neuroblastoma	45
15	3 years	M	Ptosis + restriction to elevation in adduction	Acquired Brown’s syndrome (unknown etiology)	10
16	12 years	F	Ptosis + papilledema+ superior rectus palsy	Pseudotumor cerebri	22

M= male; F= female; MFS= Miller Fisher syndrome.

Trauma was the most common cause of transient blepharoptosis, and trauma-related blepharoptosis was observed in 7 patients (43.7%), none of whom had intracranial hemorrhage. All the patients with traumatic blepharoptosis had a history of minor or mild cranial trauma. None of the patients had additional findings that could cause mechanical ptosis, such as eyelid edema, ecchymosis, or hematoma. Cranial ± orbital magnetic resonance imaging (MRI) revealed no abnormal findings in all the patients. Three patients had other ocular findings (superior rectus muscle palsy in 2 patients and inferior rectus muscle palsy in 1 patient) in addition to blepharoptosis. All the patients with traumatic cases were followed up without treatment but achieved complete resolution by a maximum of 7 days.

**Figure 1:**
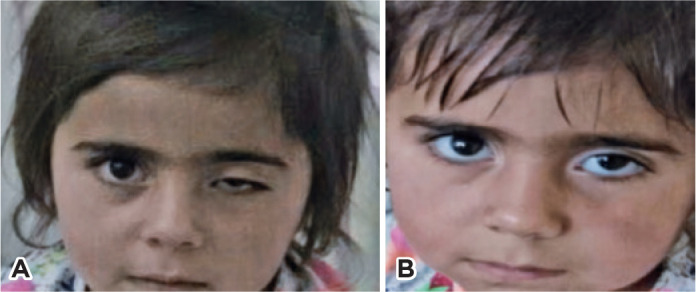
Case 7: Right ptosis in a patient with para-infectious oculomotor nerve palsy (A) and complete recovery after 6 days without treatment (B).

Infection was the second most common etiological cause of transient blepharoptosis in all the cases, and 5 (31.2%) of the 16 cases were associated with an infectious etiology. Myositis (elevated blood creatine kinase levels and myalgia) was detected in 1 patient with acute pharyngitis, and respiratory syncytial virus (RSV) was isolated (multiplex polymerase chain reaction) from the patient’s respiratory tract (nasopharyngeal aspirate test; Figure 1). In this infectious group, only 1 patient had an ocular finding (medial rectus muscle palsy) other than blepharoptosis. Complete improvement occurred by 6 days in all the infection-related blepharoptosis cases upon the regression of the infectious findings.

**Figure 2:**
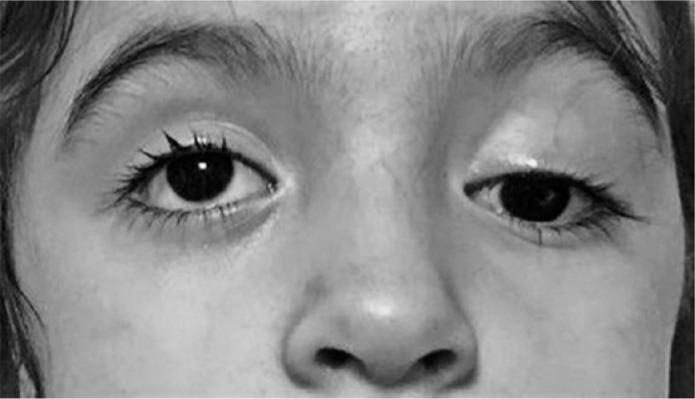
Case 13: Left-sided blepharoptosis and upward gaze palsy in a 30-month-old patient with Miller Fisher syndrome.

One patient was diagnosed as having Miller Fisher syndrome (MFS); 1 patient, Horner syndrome (HS) secondary to neuroblastoma; 1 patient, pseudotumor cerebri; and 1 patient, acquired Brown’s syndrome (unknown etiology). The patient with MFS presented with upward gaze palsy plus ataxia, reduced deep tendon reflexes, and blepharoptosis (Figure 2). Lumbar puncture and MRI were performed to diagnose MFS, and an increased protein level was detected in the cerebrospinal fluid, but the MRI revealed no abnormal findings. The findings secondary to MFS were treated with intravenous immunoglobulin (IVIG), and the blepharoptosis completely regressed after the IVIG treatment.

**Figure 3:**
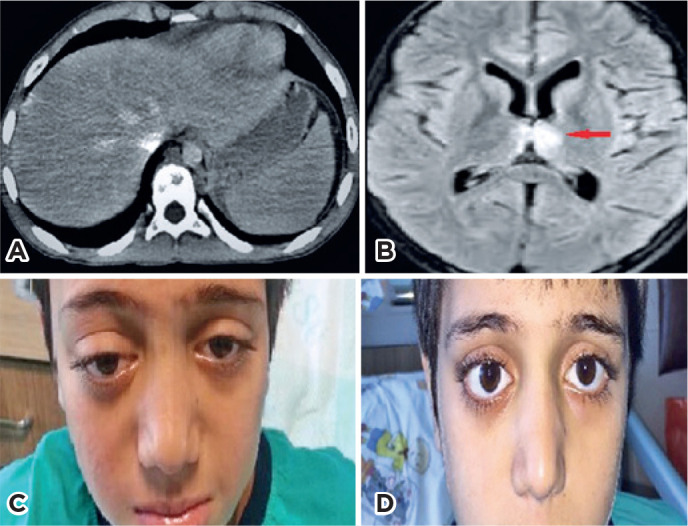
Case 14: (A) Abdominal computed tomography image showing lytic lesions on the vertebra and a mass compatible with neuroblastoma anterior to the left kidney. (B) Diencephalic metastatic lesion before chemotherapy. (C) Right-sided blepharoptosis before the chemotherapy. (D) Regression of the ocular findings after the chemotherapy.

The patient with HS was admitted with right-sided blepharoptosis and severe headache. On physical examination, papilledema and miosis were detected. MRI and computed tomography revealed a solid mass compatible with left suprarenal neuroblastoma, a metastatic intracranial (diencephalon) lesion, and numerous ly ticinfiltrative lesions in the thoracic and vertebral bones and adjacent tissues. The patient underwent surgery and chemotherapy for the treatment of the neuroblastoma, which resulted in the complete improvement of the ocular findings 45 days after the onset of the symptoms (Figure 3).

The patient with acquired Brown’s syndrome presented with sudden-onset unilateral ptosis, inability to look upward, and chin-up head position. Orbital gadolinium contrast-enhanced MRI revealed superior oblique muscle tendinitis, although cranial imaging and laboratory tests revealed no abnormal findings. The blepharoptosis regressed spontaneously without treatment on the 10th day after symptom onset. However, the restricted elevation of the left eye in the adduction position, which occurred owing to the involvement of the superior oblique muscle, persisted.

**Figure 4:**
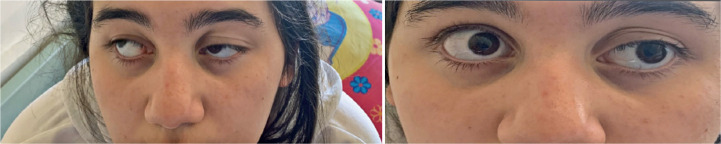
Case 16: Left-sided ptosis and upward gaze palsy in a patient with idiopathic intracranial hypertension.

A patient with idiopathic intracranial hypertension presented with ptosis, diplopia, and upward gaze palsy (Figure 4). The patient’s lumbar puncture examination revealed a CSF pressure of 35 mm/H_2_O (normally <20 mm/H_2_O in children). The patient’s symptoms improved after acetazolamide treatment.

## DISCUSSION

The acquired causes of blepharoptosis in the pediatric age group have been rarely investigated by comprehensive studies. Rasiah et al.^(4)^ indicated that infantile hemangioma, trauma, and idiopathic blepharoptosis are the most common causes of mechanical ptosis among the acquired pediatric causes of ptosis. The mechanism underlying the occurrence of transient traumatic isolated blepharoptosis after mild head trauma is not well understood. We could speculate this to be due to a transient injury/dysfunction of the ramus superior, which might not be detected on imaging. Unlike the previous study, our case series only included cases of acute-onset blepharoptosis. Therefore, we did not analyze eyelid masses that caused blepharoptosis by a mechanical effect. On the other hand, Rasiah et al. did not provide detailed information about cases cause by trauma, which is the second most common cause of blepharoptosis. This is because blepharoptosis has already been shown to be caused by not only mechanical effects of trauma but also neurogenic factors^(12,13)^. Trauma was also previously shown to be the most common cause of third cranial nerve palsy in pediatric cases^(14,15)^. In our study, the most common cause (43.7%) was trauma. Cranial imaging studies are used to eliminate certain conditions such as eyelid edema and hematoma that may potentially lead to mechanical blepharoptosis in trauma patients. We believe that trauma-associated temporary blepharoptosis may be of neurogenic origin on the basis of the presence of extraocular muscle involvement in 2 of the 7 cases with trauma-associated ptosis, the absence of lid trauma causing ptosis, and the regression of blepharoptosis by around 1 week.

Neurological complications are common in infectious diseases such as encephalitis, acute flaccid paralysis, and optic neuritis. The association between pediatric blepharoptosis related to oculomotor nerve palsy and the development of infectious diseases is not well established. The pathophysiology of transient blepharoptosis also remains unclear; it might be caused by minor inflammatory edema (as hypothesized in sinusitis/orbital cellulitis) and could also be para-infectious. It may be related with direct invasion of a virus and cytokines to neural tissues (e.g., neurons) and an indirect post-infectious immunologically mediated mechanism. Thus, it may occur as part of neuropathy. Post-viral infection blepharoptosis and partial oculomotor nerve palsy have been reported to occur in children^(16,17)^. The RSV spreads from the respiratory tracts to the central nervous system through a hematogenous route, thereby altering the local homeostasis. Upon the onset of RSV infection, the virus spreads from the lungs to the brain via the hematogenous route. Elevated levels of interleukins such as IL-6 and IL-8 have been found in CSF samples from patients with the infection, along with the detection of antibodies against the virus and viral RNA^(17)^. Our study revealed infection as the second most common cause of acute-onset acquired blepharoptosis, with a prevalence of 31.2% (n=5). Physical examination, clinical history, and laboratory findings were used to diagnose and confirm an infectious etiology. All the patients with infection-related blepharoptosis had a history of febrile infectious disease 2-6 days before the onset of ptosis. All these patients had a history of acute upper respiratory tract infection (acute tonsillitis, acute pharyngitis, etc.). None of the patients had orbital cellulitis or severe sinusitis. To our knowledge, this is the first case of oculomotor nerve palsy occurring after the onset of RSV infection.

MFS, which is considered a rare variant of Guillain- Barré syndrome, is characterized by external ophthalmoplegia, areflexia, and ataxia and plays a role in the etiology of blepharoptosis. MFS is a para-infectious phenomenon and autoimmune neuropathy that occurs after the onset of a gastrointestinal or respiratory infection^(18)^. A 30-month-old girl had ataxia, blepharoptosis, and ophthalmoplegia in her left eye. Her complaints started for 3 days before. Consecutive examinations revealed decreased deep tendon reflexes. Cerebral and spinal MRI revealed no abnormalities. An electrophysiological examination revealed no F waves, which prompted a study of antiganglioside GQ1B (anti-Gq1b) antibody. After the anti-Gq1b test returned a positive result, IVIG therapy was administered immediately at a dose of 0.4 g/kg/day (2 g/kg/total dose) for 5 days. A near-total improvement of the signs occurred on the ninth day of therapy. We believe that the five cases related to upper respiratory infections and 1 case of MFS may be broadly referred to as “para-infectious blepharoptosis.” Previous studies indicated that various cranial nerves and their branches may be involved in para-infectious causes. The possibility of a direct invasion of the neural tissue by the virus or a post-infection immune-mediated mechanism in an infection-related neural involvement has been emphasized^(11,16-19)^.

Tumoral formations commonly cause blepharoptosis by exerting a direct mechanical effect. However, albeit rare, some conditions such as neuroblastoma may cause HS by involving the mediastinum, which indicates that patients may present with atypical signs such as blepharoptosis. In HS, the lesion may occur in the cerebral hemisphere, hypothalamus, cervical spinal cord, T1 spinal root, and carotid plexus. The the lesion can cause a prolonged sympathetic sensation of pain in the eye and form from metastases. Thus, for pediatric cases of mild acquired blepharoptosis, thorough evaluations for the presence of anisocoria and HS should be performed. Acquired HS is less commonly reported in children than congenital HS^(6)^. A 12-year-old boy presented with severe headache that started 15 days before and fever that occurred within the previous 10 days. Right-sided blepharoptosis started 5 days before. The patient was found to have increased vanilmandelic acid (VMA) and neuron-specific enolase levels in laboratory tests, and CD56 (+) cells were observed in a bone marrow aspiration biopsy. MRI revealed a solid mass compatible with left suprarenal neuroblastoma, a metastatic intracranial (diencephalon) solid mass. He was diagnosed as having neuroblastoma and central HS.

Brown’s syndrome is a disorder characterized by the involvement of ocular muscles, causing abnormalities in gaze positions. Although various associations have been shown for acquired Brown’s syndrome, its mechanism is still incompletely understood. These patients rarely develop blepharoptosis, and pseudoptosis commonly develops as a result of hypotropia and may be confused with true blepharoptosis^(20-23)^. However, true blepha roptosis was diagnosed in our patient by detailed examinations performed by an ophthalmologist and a pediatric neurologist. Although the patient’s gaze limitation did not improve during the clinical follow-up or according to the family’s observation, the eyelid ptosis had improved on the 10th day of follow-up.

Idiopathic intracranial hypertension (IIH) is rare in childhood, whereas sixth nerve palsy is common among patients with IIH-related oculomotor nerve palsy. While many etiological factors are responsible for IIH, overweight has recently emerged as one of the most important risk factors. Therapeutic lumbar puncture, acetazolamide therapy, topiramate therapy, and weight loss (in obese or overweight patients) are treatment options for IIH^(24,25)^. Tan et al.^(26)^ described a girl with bilateral partial oculomotor palsy secondary to IIH with preservation of the pupillary fibers. The brain and orbital MRI examinations of our patient did not show any abnormalities. The MR venography finding was normal. Cerebrospinal fluid examination revealed no infection or any biochemical abnormality. The CSF opening pressure was 35 mm/H_2_O (normally <25 mm/H_2_O)^(27)^. All examination results, including history of medication intake, antinuclear antibody, thyroid function tests, complete blood count, biochemical studies, and vitamin A-D levels, were normal. Thyroid-stimulating hormone receptor antibody and antinuclear antibody test results were negative. On physical examination, the patient’s weight was 59.6 kg (within the 90th-97th percentiles), height was 152 cm (within the 25th-50th percentiles), and body mass index was 22.7 kg/m^2^ (within the 85th- 95th percentiles). Acetazolamide treatment and weight loss by dietary recommendations led to improvements of the symptoms.

In many cases in this series, ptosis did not occur long enough to cause ocular complications such as amblyopia. In the patient with the longest disease duration, the ptosis was due to Horner’s syndrome and did not require ocular treatment, as the upper eyelid did not cover the pupil opening. Likewise, as the findings of the patients with extraocular muscular involvement and ptosis quickly regressed, no treatment other than observation was recommended. The unavailability of certain parameters such as ptosis severity, levator muscle function, and margin reflex distance is one of the major limitations of our study.

However, the patient’s recovery time along with ptosis severity are important considerations for making therapeutic decisions. RSV was isolated in only one of the infection-related ptosis cases, and no pathological cause was identified other than clinical history and infectionrelated laboratory abnormalities in the other cases. We believe that performing diagnostic studies directed at potential causes in future studies would contribute to the literature.

In conclusion, acute-onset unilateral acquired ptosis in the pediatric age group is characterized by a spontaneously terminating course that requires no surgical intervention. However, para-infectious causes such as MFS and GBS, and malignancies such as neuroblastoma may cause blepharoptosis. Such patients may need agent-specific treatments even if they do not require eyelid surgery. This study shows that the most common causes of acute-onset, transient unilateral acquired ptosis in the pediatric population are trauma and infection. It also emphasized that apart from these causes, MFS, neuroblastoma, pseudotumor cerebri, and acquired Brown’s syndrome may also cause blepharoptosis.
